# Positron emission tomography imaging of coronary atherosclerosis

**DOI:** 10.2217/fca-2016-0017

**Published:** 2016-06-20

**Authors:** Alastair J Moss, Philip D Adamson, David E Newby, Marc R Dweck

**Affiliations:** 1 Centre for Cardiovascular Science, University of Edinburgh, Edinburgh, UK; 2 Translation Molecular Imaging Institute, Icahn School of Medicine at Mount-Sinai, NY, USA

**Keywords:** atherosclerosis, cardiac imaging, personalized medicine

## Abstract

Inflammation has a central role in the progression of coronary atherosclerosis. Recent developments in cardiovascular imaging with the advent of hybrid positron emission tomography have provided a window into the molecular pathophysiology underlying coronary plaque inflammation. Using novel radiotracers targeted at specific cellular pathways, the potential exists to observe inflammation, apoptosis, cellular hypoxia, microcalcification and angiogenesis *in vivo*. Several clinical studies are now underway assessing the ability of this hybrid imaging modality to inform about atherosclerotic disease activity and the prediction of future cardiovascular risk. A better understanding of the molecular mechanisms governing coronary atherosclerosis may be the first step toward offering patients a more stratified, personalized approach to treatment.

**Figure 1.**
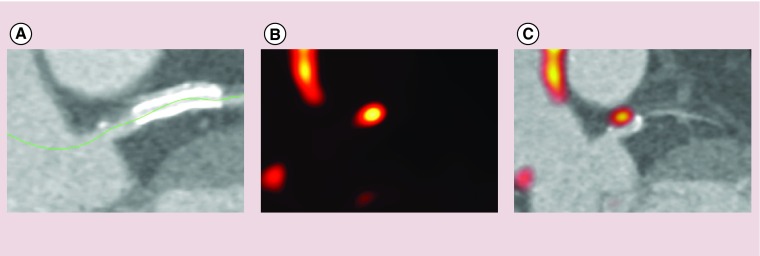
**^18^F-sodium fluoride and plaque rupture.** Patient presenting with acute myocardial infarction. **(A)** Computed tomography demonstrating high-risk features (spotty calcification and low attenuation plaque) in that region. **(B & C)** The culprit plaque also demonstrated increased ^18^F-fluoride positron emission tomography activity on hybrid positron emission tomography/CT image.

**Figure 2.**
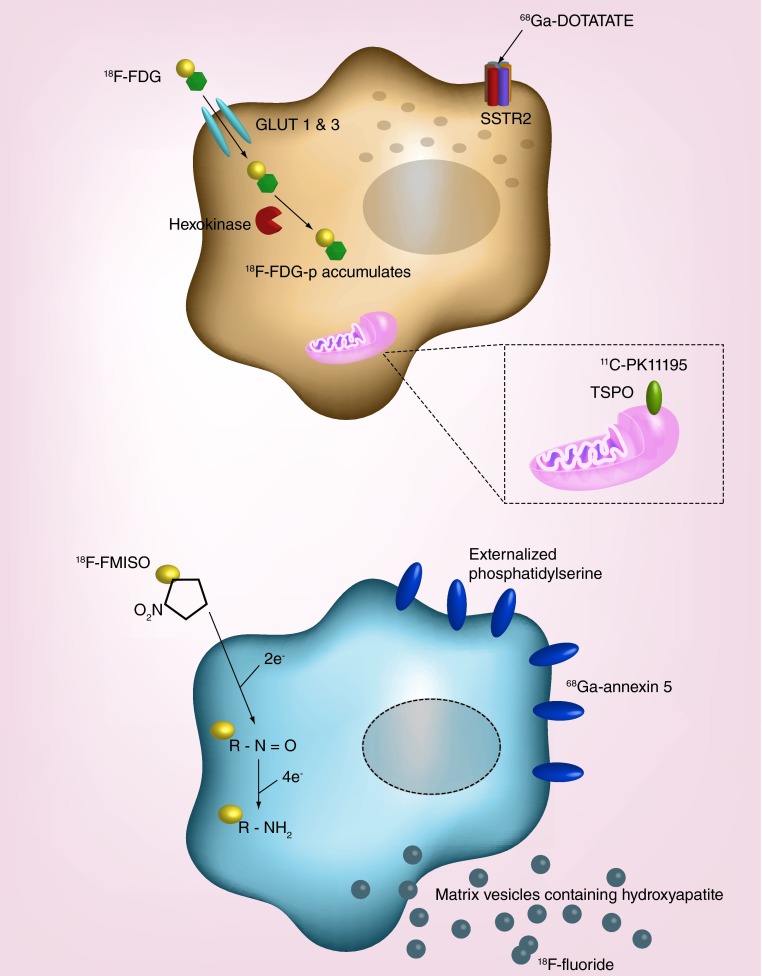
**Radiotracer accumulation in atherosclerotic plaques as markers of inflammation, hypoxia, apoptosis and microcalcification activity.** Inflammatory pathways can be visualized *in vivo* using specific radiolabeled positron emission tomography ligands. ^18^F-FDG accumulates in activated macrophages, but can also be influenced by local hypoxia. Other radiotracers, such as ^68^Ga-DOTATATE and ^11^C-PK11195, may be more specific markers of macrophage activity than ^18^F-FDG. An imbalance in metabolic substrates reduces the oxygen tension in the plaque promoting necrosis and apoptosis of macrophages and smooth muscle cells. Generation of reactive oxygen species, externalization of phosphatidylserine and the extrusion of microcrystalline hydroxyapatite can be detected by ^18^F-FMISO, ^68^Ga-Annexin 5 and ^18^F-fluoride, respectively. FDG: Fluorodeoxyglucose; FDG-p: Fluorodexoyglucose-6-phosphate; FMISO: Fluoromisonadazole.

**Figure 3.**
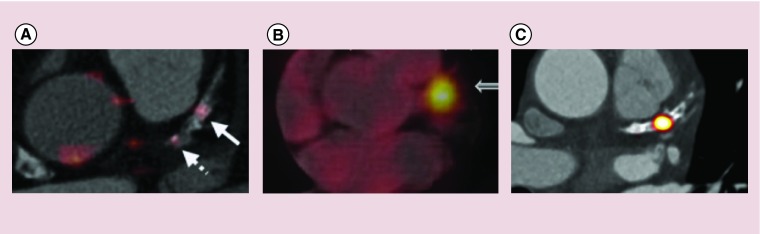
**Coronary radiotracer uptake in calcified proximal left anterior descending arteries.** Comparison of different radiotracers in the proximal left anterior descending artery of three different patients. The relatively low diffuse 2-(^18^F)-fluoro-2-deoxy-d-glucose signal **(A)** contrasts with enhanced focal uptake of ^68^Ga-DOTATATE (modified with permission from [22]) **(B)** and ^18^F-fluoride (reproduced with permission from [55]) **(C)**. Individual radiotracers can discriminate between the upregulation of different molecular pathways in macroscopically similar plaques.

Advances in cardiovascular molecular imaging offer the potential to unravel the complex *in vivo* cellular pathophysiology of a variety of cardiovascular disorders. Through a greater understanding of the pathogenesis of cardiovascular disease, molecular imaging may highlight novel risk factors and key targets for future treatments. Whereas traditional structural imaging modalities yield a generic approach to patient care, molecular imaging modalities give complementary information at the cellular level, informing about disease activity with the potential to facilitate personalized disease monitoring and therapy.

Atherosclerosis is a chronic inflammatory disease characterized by the formation of lipid-rich plaques. As early as the mid-19th century, Ruldoph Virchow advocated the role inflammation plays in the formation of Grützbalg (grit follicles) within the coronary vasculature, namely that ‘inflammation of the inner arterial coat be the starting point of atheromatous degeneration’ [1]. Our understanding of the pro-inflammatory mechanisms associated with atherosclerotic events subsequently expanded through pathological studies of autopsy specimens and preclinical models [2–5]. These demonstrated inflammation to be a central process at almost every stage of atherosclerosis but particularly important in the precipitation of acute plaque rupture, with macrophages secreting matrix metalloproteinases that serve to weaken the fibrous cap. Inflammation is, therefore, a key feature of plaques at risk of rupture in addition to other related characteristics, such as a large necrotic core, a thin fibrous cap, angiogenesis and microcalcification. Each represents a potential imaging target, with molecular imaging well placed to measure noninvasively the *in vivo* activity of these key processes.

The emergence of hybrid coronary positron emission tomography (PET) in conjunction with computed tomography (CT) or magnetic resonance offers the opportunity to investigate pathophysiological processes on a molecular scale. Potentially any pathological feature can be targeted depending on the availability of an appropriate radiopharmaceutical. Current approaches have made use of established radiotracers developed for oncological imaging. However, the potential exists to create bespoke tracers targeted specifically at high-risk atherosclerotic plaque features, thereby providing even more accurate imaging. This review will investigate current and emerging PET tracers that hold promise in resolving the activity of these key processes underlying events and the progression of atherosclerosis in humans. It will also examine some of the technological challenges that will need to be met in order to translate these approaches in to the coronary arteries.

## ^18^F-fluorodeoxyglucose & macrophage metabolism

2-(^18^F)-fluoro-2-deoxy-d-glucose (^18^F-FDG) is a glucose analog that enters cells expressing glucose transporters (known as solute carrier family 2, facilitated glucose transport member [GLUT] 1 and 3) by facilitated diffusion. It accumulates in the cytosol following phosphorylation by hexokinase to ^18^F-FDG-6-phosphate. The stoichiometry of ^18^F-FDG-6-phosphate prohibits further breakdown via the glycolytic pathway leading to a rise in concentration of ^18^F-FDG-6-phosphate that is proportional to the metabolic demand of the cell [6]. Macrophages are the key cellular constituent of active, inflamed atheroma and have increased metabolic demands compared with surrounding cells in the vasculature. *Ex vivo* studies have demonstrated a close histological correlation between ^18^F-FDG uptake and macrophage density [7]. Macrophage density correlates with plaque progression and the size of the necrotic core [8,9]. Preclinical mouse models suggest that ^18^F-FDG may signal inflammation during the early phase of atherogenesis at the point of foam cell formation, while clinical studies have suggested uptake is less evident once macroscopic calcium has formed within the plaque [10,11]. Satomi *et al*. reported that the polarization of macrophages toward a pro-inflammatory M1 subtype results in increased tritiated fluorodeoxyglucose (^3^H-FDG) uptake through the upregulation of glucose transporters compared with a reparative M2 subtype [12]. A two-fold increase in ^3^H-FDG was associated with an increase in GLUT-1 and 3 and hexokinase gene expression in M1 macrophages. Additionally, there was downregulation of glucose-6-phosphorylase, the reverse reaction of hexokinase. The same was not observed in M2 macrophages, which instead appear to decrease their dependence on glycolysis, perhaps in order to gain a survival advantage in the hostile inflammatory environment [13].

The pivotal work of Rudd *et al*. demonstrated that ^18^F-FDG uptake can be visualized in the atheromatous walls of the carotid artery [14]. In eight patients with symptomatic carotid lesions, there were higher estimated ^18^F-FDG accumulation rates compared with asymptomatic lesions and no significant uptake in angiographically normal arteries. The carotids are especially well suited to vascular PET imaging because of their relatively large caliber, stationary nature and because vascular tissue is readily available for histological validation following endarterectomy. Optimization of scanning protocols to determine the appropriate injected dose, circulation time and prescan fasting glucose have been conducted allowing quantification of carotid ^18^F-FDG with excellent scan-rescan reproducibility [15–18]. This means that relatively few patients are required for clinical trials testing the anti-inflammatory effects of novel medication. This has facilitated the use of FDG–PET as a surrogate end point in Phase II clinical trials of novel pharmaceuticals, with the results closely monitoring the outcomes of subsequent studies focusing on clinical end points [19–21]. Whether this approach will expedite the translation of drug discovery toward successful Phase III trials remains to be seen with several Phase IIa studies using ^18^F-FDG uptake as a primary end point due to report later this year.

Translating ^18^F-FDG PET in to the coronary arteries is more challenging owing to the limited spatial resolution of clinical PET platforms and the impact of coronary motion. Assessment of the proximal coronary arteries and aortic root appears feasible with increased uptake observed in patients with recent acute coronary syndrome [22]. However, more detailed analysis of the mid and distal coronary vasculature is limited by spillover of signal from the myocardium, such that even with optimal myocardial suppression protocols, half of all coronary territories cannot be interpreted [23,24]. Clinical studies examining alternative methods for reducing physiological ^18^F-FDG uptake in the myocardium are under investigation in the hope that they will improve visualization of coronary activity. Combined with the multitude of factors that may account for increased ^18^F-FDG uptake, these limitations have shifted attention to find more specific radiotracers that target inflammatory pathways, however none of these have made it through to clinical application to date.

## ^18^F-FDG & biomarkers of inflammation

The association between ^18^F-FDG uptake and other biomarkers of inflammation offers insight into the complex processes governing atherosclerotic progression. Following the publication of the JUPITER trial, interest in evaluating the inflammatory state of atherosclerotic plaques continues to grow [25]. The use of high sensitivity C-reactive protein provides a snapshot of inflammatory activation within the body; however, it cannot localize this activity to the vasculature let alone specific vascular territories or plaques. By contrast, colocalization of ^18^F-FDG to the arterial wall gives a clearer signal of focal atherosclerotic inflammation. Unraveling the complex relationship between systemic inflammation and ^18^F-FDG uptake is challenging, especially as plaque rupture events following myocardial infarction may exacerbate atherosclerotic inflammation at remote sites [26,27]. Importantly, ^18^F-FDG plaque imaging appears more sensitive in detecting the anti-inflammatory effects of novel atherosclerosis therapies making it a useful end point for Phase II clinical trials [20,28]. From a clinical standpoint, identifying ^18^F-FDG uptake within carotid arteries improves cardiovascular risk stratification independent of high sensitivity C-reactive protein suggesting that ^18^F-FDG offers incremental information to the assessment of atherosclerotic inflammation [29].

Preclinical ^18^F-FDG imaging has provided a deeper understanding of plaque pathophysiology and temporal fluctuations in plaque activity. Murine models using ApoE^-/-^ mice fed on a high-fat western diet exhibit higher ^18^F-FDG uptake within the descending aorta at 16 weeks. Lipid-rich plaques that display focal regions of ^18^F-FDG uptake recruit macrophages through the expression of VCAM-1. Associations between plaque inflammation and subsequent calcification have also been observed using ^18^F-FDG. ^18^F-FDG highlights regions of the vasculature associated with osteopontin, a marker of early vascular calcification, which has prognostic significance in human coronary atherosclerosis [30]. High osteopontin levels are associated with increased major adverse cardiovascular events following myocardial infarction, stable coronary artery disease and in patients undergoing coronary intervention [31–33]. Over 5-years follow-up, focal ^18^F-FDG uptake identified locations of subsequent calcium deposition and calcium progression in the thoracic aorta. While plaques with novel calcification were associated with increased arterial inflammation, regions of dense calcification were associated with a decreased level of inflammatory activity [34,35]. Understanding the exact link between inflammation and calcification in atherosclerosis is a key topic of investigation, which may be aided by ^18^F-fluoride PET imaging and is discussed in greater detail below.

## Link between inflammation & calcification

Vascular calcification is a key process in atherosclerosis although its exact role remains incompletely understood. Large macroscopic calcific deposits can be visualized on CT and are usually associated with advanced stable atherosclerotic plaque. Nevertheless, CT-determined calcium predicts future adverse events presumably on the basis that patients with high calcium scores will also have more noncalcific plaques at risk of rupture and causing an event. By contrast, the early stages of calcification appear to be associated with plaque instability and increased risk of rupture and events. Ruptured and culprit plaques often demonstrate regions of microcalcification on histopathology. In keeping with this hypothesis low-density calcium deposition on CT is associated with an increased risk of events compared with high-density calcium [36]. Similarly, the early stages of ‘spotty’ macrocalcification on CT appear to mark out a higher risk stage of the disease than the larger macroscopic deposits found in stable plaques [37]. CT imaging however, is unable to resolve true microcalcification, prompting investigation of alternative approaches with the capacity to detect this early form of calcium.

The association between plaque inflammation and subsequent calcification has been studied using molecular imaging in preclinical models of atherosclerosis. High-resolution molecular imaging with a bisphosphonate-derived near-infrared imaging agent (OsteoSense) identifies distinct regions of calcification activity in the vasculature that can be compared with macrophage staining [38]. Indeed, OsteoSense binds preferentially to nascent, nanocrystalline deposits of hydroxyapatite that coalesce to form spheres of microcalcification of <50 μm. Microscopic analysis of these particles reveals a range in size from 5 μm down to individual matrix vesicles that are only 100 nm [39]. Cell-derived matrix vesicles containing nanocrystals of hydroxyapatite are exocytosed from the cell membranes of macrophages and vascular smooth muscle cells. These are then released into the extracellular space where they form a nidus for microcalcification, which then aggregate to form larger macroscopic deposits [40,41]. This process is most evident in the necrotic core where nucleation and calcification growth occur in regions of collagen degradation [41]. Interestingly, hydroxyapatite may also be a contributor to the inflammatory process, rather than merely an end product. Ectopic needle-shaped hydroxyapatite crystals stimulate IL-1β/IL-18-dependent inflammatory pathways [42]. Additionally, if pairs of microcalcified spheres migrate into the fibrous cap, increases in local mechanical stresses due to interfacial debonding can lead to plaque rupture [43].

## ^18^F-sodium fluoride & microcalcification

(^18^F)-sodium fluoride (^18^F-fluoride) has been used as an oncological radiotracer for the past 40 years. ^18^F-Fluoride binds to hydroxyapatite by substitution with a hydroxyl group on the surface of the hydroxyapatite matrix to form fluoroapatite (Ca_10_[PO_4_]_6_F_2_) [44]. As hydroxyapatite is the dominant *in vivo* form of crystalline calcium, ^18^F-fluoride has been used to define areas of increased bone activity with greater accuracy in detecting bone metastases than other conventional imaging techniques [45].

Hydroxyapatite is also the major component of vascular calcification leading to interest in using ^18^F-fluoride as a marker of vascular calcification activity. Early studies demonstrated areas of increased PET uptake in the aorta, the valves of patients with aortic stenosis and the coronary arteries [24,46–49]. The latter is of particular interest with ^18^F-fluoride localizing to individual plaques with excellent S/N and very low uptake in the surrounding myocardium [50,51].

^18^F-fluoride appears to provide different information to CT calcium scoring. Detailed electron microscopic analysis of carotid endarterectomy specimens has shown that fluoride, like OsteoSense, binds preferentially to regions of microcalcification compared with the macroscopic deposits observed using CT [52]. This perhaps reflects the greater exposed surface area of hydroxyapatite in these nanocrystalline areas [52]. Indeed, 41% of patients with coronary artery calcium scores >1000 do not have any evidence of increased ^18^F-fluoride uptake [24]. As previously discussed, microcalcification is a key component of high-risk atherosclerotic plaques and consistent with this, plaques with increased ^18^F-fluoride uptake have been shown to have multiple high-risk characteristics on histology and intravascular ultrasound including inflammation, positive remodeling, microcalcification and a large necrotic core (Figure 1). Moreover, in patients with recent myocardial infarction increased ^18^F-fluoride uptake has been observed in >90% of the culprit plaques responsible for that event [23]. The clinical challenge is to now determine whether identification of microcalcification in the coronary arteries using ^18^F-fluoride PET can improve risk stratification in individuals at high risk of future coronary thrombotic events [53]. This hypothesis forms part of the on going PRE^18^FFIR study (ClinicalTrials.gov: NCT02278211). Briefly, this multicenter PET study will recruit 700 participants with multivessel coronary artery disease and recent myocardial infarction to perform ^18^F-fluoride imaging to evaluate whether increased coronary uptake can predict future cardiovascular events over 2-years follow-up.

## Developments in coronary artery PET

The development and expansion of PET will depend on the development of novel tracers targeting a range of different pathological processes (Figure 2). A number of tracers are in development with many centered on providing more specific imaging of inflammation, while others have been targeted to related processes associated with active atheroma, disease progression and plaque rupture (Table 1). In addition, improvements in spatial resolution and techniques for correcting cardiac motion will be required if these new tracers are to prove of use in the small and highly mobile coronary arteries.

## Novel tracers

### • ^68^Ga-DOTATATE & macrophage somatostatin receptors

Somatostatin receptors are G-protein-coupled receptors that are expressed in a wide variety of tissues. ^68^Ga-DOTATATE binds to SSTR2, which are expressed by the lipopolysaccharide-activated macrophages associated with plaque vulnerability [67]. Whereas ^18^F-FDG is hampered by diffuse myocardial uptake that often obscures coronary activity, ^68^Ga-DOTATATE permits clearer detection of macrophage accumulation in coronary plaques (Figure 3). In a retrospective analysis in 70 patients with neuroendocrine tumors, 44% had colocalization of ^68^Ga-DOTATATE to atheromatous plaques in the proximal coronary arteries [54]. While in another retrospective study of 44 cancer patients ^68^Ga-DOTATATE again accumulated in individual coronary lesions suggesting that it may ultimately prove a better marker of inflammation activity in these vessels that ^18^F-FDG [55]. This is the subject of the ongoing prospective VISION study (ClinicalTrials.gov: NCT02021188).

### • ^11^C-PK11195 & translocator protein/peripheral benzodiazepine receptors

(^11^C)-PK11195 is an isoquinoline-derived ligand of the TSPO, previously known as the peripheral benzodiazepine receptor that is found on the outer mitochondrial membrane. TSPO is involved in cholesterol transport across the mitochondrial intermembrane space and regulation of the mitochondrial respiratory chain. This critical role means that TSPO is widely expressed in cardiac tissues, however, the highest density of receptors are found in activated macrophages undergoing bursts of oxidative stress [68,69]. In a proof of concept study, Pugliese *et al*. visualized ^11^C-PK11195 uptake in six patients with large vessel vasculitis in the aortic arch and carotid arteries [70]. Symptomatic patients with active disease had higher signals in the vascular wall compared with asymptomatic patients with quiescent vasculitis. The same group performed ^11^C-PK11195 imaging in patients with carotid atherosclerosis demonstrating increased tracer uptake in the ipsilateral culprit carotid plaque of patients post stroke/transient ischemic attack (TIA) [56]. In eight patients undergoing carotid endarterectomy, *ex vivo* autoradiography using ^3^H-PK11195 confirmed radiotracer colocalization with CD68^+^ macrophages. While encouraging, wider application of ^11^C-PK11195 may prove limited due to variance in expression of a common polymorphism in the TSPO gene (rs6971) that affects the binding affinity of ligands and the quantification of TSPO derived PET tracers in approximately 30% of Caucasians [71]. Larger prospective clinical outcome studies will need to account for the frequency of TSPO polymorphisms in the population if accurate *in vivo* measurements are to be used for diagnosis and monitoring of therapeutic effects [57].

### • ^18^F-fluoro-d-mannose & M2 macrophages

Mannose, an isomer of glucose, also serves as a substrate for glycolysis in metabolically active macrophages. Similar to glucose, it is incorporated into cells through glucose transporters, but also binds to mannose receptors expressed on M2 macrophages [72]. As such, radio labeled 2-deoxy-2-(^18^F)fluoro-d-mannose (^18^F-FDM) has been explored as a viable alternative to ^18^F-FDG for imaging inflammation in atherosclerosis [59]. This preclinical study highlighted an improved pharmacokinetic profile using ^18^F-FDM compared with ^18^F-FDG, which resulted in higher levels of ^18^F-FDM uptake in macrophages, predominantly due to less inhibition of hexokinase activity than is observed with ^18^F-FDG. Clinical translation of these results is now awaited.

Detection of M2 macrophages may serve as an indirect measure of plaque hemorrhage, since macrophage clearance of intracellular iron and hemoglobin generates an M2 subtype characterized by high mannose receptor expression [72]. Intraplaque hemorrhage results in rapid expansion of the necrotic core following the sudden release of cholesterol-rich erythrocyte membranes. It is therefore an important contributor to episodic plaque growth and may account for the sudden transformation of stable coronary artery disease to active disease state at increased risk of rupture [73].

### • ^18^F-fluoromisonidazole & hypoxia

Hypoxia is a key feature of both the expanding necrotic core and atherosclerotic plaque growth. In early plaques oxygen freely diffuses across the initima and the adventitial *vasa vasorum*. However with plaque expansion, this oxygen diffusion falls [74]. Combined with the increasing metabolic demand from activated macrophages, an oxygen debt builds up that renders these advanced atherosclerotic plaques severely hypoxic. ^18^F-FDG may provide an indirect measure of oxygen sufficiency as expression of reactive oxygen species and hypoxia inducible factors stimulate ^18^F-FDG uptake [75,76]. However, radiolabeled nitroimidazoles offer greater specificity as they accumulate in tissues that lack oxygen, acting as an electron carrier in the mitochondrial respiratory chain. Uptake of nitroimidazoles is inversely proportional to the oxygen tension, such that 3H-fluoromisonidazole uptake increases by 20 fold at low partial pressures of oxygen [77]. ^18^F-fluoromisonidazole (^18^F-FMISO) has been used extensively in tumor imaging and is now under investigation in atherosclerosis [62]. In a preclinical study, ^18^F-FMISO uptake colocalized with pimonidazole defined regions of hypoxia that were nestled in deep in macrophage-dense cores. By contrast, superficial macrophages in a subintimal location were not hypoxic and less inflamed. Clinical studies with a similar nitroimidazole analog ([^18^F]-2-(4-((2-nitro-1H-imidazol-1-yl)methyl)-1H-1,2,3-triazol-1-yl)propan-1-ol) have recently been reported demonstrating increased uptake in patients with carotid stenoses [63].

### • ^18^F-fluciclatide & α_V_β_3_ integrin receptors

Angiogenesis occurs in response to atherosclerotic plaque hypoxia, with fragile microvessels sprouting from the adventitial *vasa vasorum* to provide the necrotic core with a new blood supply. These thin-walled vessels have poor structural integrity and are prone to leakage, rupture and ultimately hemorrhage in to the plaque [78]. The vascular endothelial cells responsible for establishing this microvasculature express α_V_β_3_ integrin, part of the integrin superfamily of heterodimeric receptors responsible for cell adhesion and signaling. The α_V_β_3_ receptor is upregulated in immature endothelial cells as a response to angiogenic guidance molecules. The arginine–glycine–aspartate (RGD) motif has allowed investigators to target the α_V_β_3_ binding site to inhibit atherosclerotic progression [79]. ^18^F-Fluciclatide successfully visualizes tumor angiogenesis and can detect treatment responses to chemotherapy [80,81]. Preclinical experiments with a similar analog, ^18^F-RGD-K5, demonstrated a moderate correlation between PET uptake and endothelial cell staining of *ex vivo* carotid plaques [65]. Our group has recently performed the first prospective clinical study (ClinicalTrials.gov: NCT01813045) using ^18^F-fluciclatide to assess aortic atherosclerotic uptake, with data due to be reported shortly [64].

### • ^68^Ga-annexin A5 & macrophage apoptosis

The lipid-rich necrotic core is a key feature of the vulnerable plaque and is derived from the death of macrophages and smooth muscle cells within the plaque due to a combination of apoptosis and necrosis. Externalization of phosphatidylserine onto the extracellular surface of the plasma membrane is an almost universal feature of apoptosis. This makes it a useful target for detection using annexin-based radiotracers [82].

Clinical SPECT imaging of atherosclerotic apoptosis using ^99m^Tc-annexin A5 was first performed by Kietselaer *et al*. in four patients prior to carotid endarterectomy. In two patients who had suffered a recent TIA, the ipsilateral carotid artery showed annexin A5 uptake [83]. Interestingly, the other two patients with remote TIAs (3–4 months) had no observable annexin A5 uptake. Histology demonstrated a correlation between annexin A5 binding and both macrophage staining and intra-plaque hemorrhage. Development of the PET tracer ^68^Ga-annexin A5 has demonstrated promise in murine models of myocardial infarction. However, translation into clinical studies has been delayed because of sub-optimal pharmacokinetics with accumulation in the liver and kidneys [61]. Interestingly, annexin A5 also colocalizes with matrix vesicles containing hydroxyapatite in atherosclerosis, indicating that cell death may be an important trigger to microcalcification [40].

## Improving image quality

### • Technical aspects of coronary PET imaging

Clinical application of PET imaging to coronary atherosclerosis will require technical improvement with respect to spatial resolution and motion correction. Clinical PET has a fundamental limit of spatial resolution in the range of 4–5 mm for ^18^F-radiotracers. This means assessment of the coronary arteries with a luminal diameter of 2–5 mm is at the very limit of the capabilities of current clinical PET systems. This is a particular problem because of the complex motion of the coronary vessels, which can displace the right coronary artery by as much as 20 mm [84]. Visualizing and quantifying radiotracer uptake on this scale is additionally confounded by partial volume effects. Namely, the signal increases when surrounded by areas of high activity (e.g., myocardium with ^18^F-FDG) and attenuates when surrounded by areas of low activity (e.g., the lung with most tracers) [85]. This combination of image blurring (limited spatial resolution) and partial voluming (distribution of signal across voxels) can result in discrepancy between fused PET and CT data sets, requiring careful coalignment to delineate coronary artery segments with increased radiotracer uptake. ECG gating of the PET data can help substantially, but necessitates discarding much of the data resulting in reduced counts and noise. Cardiac motion correction algorithms that make use of all of the data are emerging and appear to improve the accuracy of coregistration [86,87]. A recent feasibility study has found that these algorithms can improve signal detection in the coronary arteries by a third, with particular reductions in the blurring of the PET image and background noise [88].

### • PET magnetic resonance

Hybrid PET is now being explored in conjunction with magnetic resonance (MR) platforms. Given that radiotracer uptake resides in the vessel wall rather than the coronary lumen, PET/MR can potentially provide additional information on soft tissue characterization, of particular use in assessing carotid plaque characteristics [89]. Ripa *et al*. performed the first feasibility study of carotid atherosclerotic imaging using ^18^F-FDG PET/MR in six patients without flow-limiting luminal stenosis [90]. There was a strong correlation between PET/MR and PET/CT measurements in the absence of significant disease. While the attenuation corrected CT and magnetic resonance maps differ across the two platforms, quantification of radiotracer uptake appears to be comparable [91]. Enthusiasm for extending the use of PET/MR to assessment of the coronary vasculature in high-risk groups is tempered by the temporal and spatial resolution required for coronary imaging and compounded by artifact from coronary stenting. However reliable imaging of the proximal vessels using MRA is now possible and one major potential advantage is the ability to continuously monitor cardiac motion using magnetic resonance, which can then be used to correct the PET data. In addition, radiation exposure can potentially be reduced by more than half. Studies are currently underway to further explore the utility of cardiac PET/MR imaging (ClinicalTrials.gov: NCT01418313).

## Conclusion & future perspective

While an increasing number of radiotracers are in development to specifically evaluate coronary atherosclerosis, what steps are required to take PET imaging in to the primetime of non-invasive coronary imaging? For aspirations to become a clinical reality, we believe the next decade of coronary artery PET research should focus on addressing three key objectives. First, can PET imaging identify individuals at risk of future cardiovascular events? Following the model established by other noninvasive imaging modalities, clinical outcome studies should evaluate whether PET imaging can stratify individuals at the greatest risk of myocardial infarction or cardiovascular death in primary and secondary disease settings. Second, can PET imaging assess an individual's response to therapy? Identifying whether individuals are responders or nonresponders to treatment is a perceived strength of molecular imaging and this may allow clinicians to ‘bridge the gap’ toward the delivery of personalized medicine, particularly when integrated with genetic profiling. Finally and possibly the highest bar for any imaging test to attain, can PET imaging assist in the selection of appropriate therapy to improve cardiovascular outcomes and save lives? This is likely to require randomized controlled trials testing whether the addition of molecular imaging can improve current paradigms. Ultimately if PET imaging is to have a clinical role this is likely to be in refining risk stratification in those already felt to be at risk.

Hybrid PET provides insight into the pathophysiology of atherosclerosis that until recently has been available only through autopsy examinations. The rapidly expanding number of radiotracers for the assessment of atherosclerosis now allows us to measure directly the disease activity and to evaluate the molecular mechanisms governing plaque progression and rupture. Translating this information into the clinic may ultimately provide a more stratified approach to risk prediction, ensuring that effective treatment is directed appropriately to those with active atherosclerosis, who are most likely to gain benefit. However considerable work remains to test this important hypothesis.

**Table 1.** **Positron emission tomography radiotracers for coronary atherosclerosis.**

**Target**	**Ligand**	**Radiotracer**	**Application to date**	**Ongoing clinical trials**	**Ref.**
Macrophage activation	GLUT (1 & 3) and conversion by hexokinase to ^18^F-FDG-6-phosphate	^18^F-FDG	Prospective *in vivo* studies in extracardiac atherosclerosis		[14,16,22]
			Myocardial suppression required to evaluate coronary arteries		
	Somatostatin receptor subtype 2	^68^Ga-DOTATATE	Retrospective *in vivo* studies in coronary artery disease	VISION study (ClinicalTrials.gov: NCT02021188)	[54,55]
	Translocator protein 18-kDa	^11^C-PK11195	Prospective *in vivo* study in carotid stenosis		[56]
	Translocator protein 18-kDa	^11^C-PBR28	Clinical studies in healthy controls and multiple sclerosis	Cardiac Sarcoidosis (ClinicalTrials.gov: NCT02017522)	[57,58]
	Mannose receptor	^18^F-FDM	Preclinical cell culture model		[59]
	Choline kinase phosphorylated to phosphatidylcholine	^18^F-choline	Preclinical murine model	PARISK study (ClinicalTrials.gov: NCT01899014)	[60]
Apoptosis	Phosphatidylserine	^68^Ga-annexin A5	Preclinical murine model		[61]
Hypoxia	Reduction to amine derivative in low O_2_ environment	^18^F-FMISO	Preclinical murine model		[62]
	Reduction to amine derivative in low O_2_ environment	^18^F-HX4	Proof of concept in carotid atherosclerosis		[63]
Microcalcification	Hydroxyapatite	^18^F-fluoride	Prospective *in vivo* studies in coronary and extracardiac atherosclerosis	DIAMOND study (ClinicalTrials.gov: NCT02110303)	[23,24]
				PRE ^18^FFIR study (ClinicalTrials.gov: NCT02278211)	
Angiogenesis	α_V_β_3_ and α_V_β_5_ integrin	^18^F-fluciclatide	Proof of concept in aortic atherosclerosis	Angiogenesis and Fibrosis in Myocardial Infarction (ClinicalTrials.gov: NCT01813045) and Aortic Stenosis (ClinicalTrials.gov: NCT01837160)	[64]
	α_V_β_3_ integrin	^18^F-RGD-K5	*Ex vivo* human carotid studies	Carotid Plaque Imaging Study (ClinicalTrials.gov: NCT01968226)	[65]

FDG: Fluorodeoxyglucose; FMISO: Fluoromisonadazole; RGD: Arginine–glycine–aspartate.

Data taken with permission from [66].

**EXECUTIVE SUMMARY**

**Inflammation & coronary atherosclerosis**

Macrophage infiltration is a key precipitant of atherosclerotic plaque rupture and adverse cardiovascular events.
Activated macrophages secrete matrix metalloproteinases that weaken fibrous caps rendering atherosclerotic plaques prone to rupture.

**Markers of macrophage activation**

2-(^18^F)-fluoro-2-deoxy-d-glucose is a positron emission tomography (PET) tracer and glucose analog that is taken up cells with high metabolic requirements including vascular macrophages.
Although 2-(^18^F)-fluoro-2-deoxy-d-glucose PET has become a useful marker of vascular inflammation in the carotid arteries and aorta, physiological uptake by the myocardium currently limits its use in the coronary arteries.
More specific markers of macrophage activation including ^68^Ga-DOTATATE and ^11^C-PK11195 are being developed.

**^18^F-sodium fluoride & microcalcification**

Microcalcification is associated with high-risk atherosclerotic plaques and is believed to form in response to cell death and inflammatory processes within these lesions.
^18^F-fluoride binds preferentially to areas of vascular microcalcification, localizing to culprit and high-risk plaques in the coronary vasculature.

**Technical improvements in coronary PET imaging**

Motion correction algorithms improve the evaluation of radiotracer uptake in coronary arteries.
PET/magnetic resonance potentially allows for improved soft tissue characterization of atherosclerotic plaques in the carotid arteries and in the coronaries may facilitate enhanced motion correction while reducing radiation exposure.

**Conclusion & future perspective**

Hybrid PET can be used to observe the molecular pathways involved in coronary atherosclerosis and as a marker of disease activity.
